# Doxorubicin in Combination with a Small TGFβ Inhibitor: A Potential Novel Therapy for Metastatic Breast Cancer in Mouse Models

**DOI:** 10.1371/journal.pone.0010365

**Published:** 2010-04-28

**Authors:** Abhik Bandyopadhyay, Long Wang, Joseph Agyin, Yuping Tang, Shu Lin, I-Tien Yeh, Keya De, Lu-Zhe Sun

**Affiliations:** 1 Department of Cellular and Structural Biology, University of Texas Health Science Center, San Antonio, Texas, United States of America; 2 Department of Biochemistry, University of Texas Health Science Center, San Antonio, Texas, United States of America; 3 Department of Pathology, University of Texas Health Science Center, San Antonio, Texas, United States of America; The Beatson Institute for Cancer Research, United Kingdom

## Abstract

**Background:**

Recent studies suggested that induction of epithelial-mesenchymal transition (EMT) might confer both metastatic and self-renewal properties to breast tumor cells resulting in drug resistance and tumor recurrence. TGFβ is a potent inducer of EMT and has been shown to promote tumor progression in various breast cancer cell and animal models.

**Principal Findings:**

We report that chemotherapeutic drug doxorubicin activates TGFβ signaling in human and murine breast cancer cells. Doxorubicin induced EMT, promoted invasion and enhanced generation of cells with stem cell phenotype in murine 4T1 breast cancer cells in vitro, which were significantly inhibited by a TGFβ type I receptor kinase inhibitor (TβRI-KI). We investigated the potential synergistic anti-tumor activity of TβR1-KI in combination with doxorubicin in animal models of metastatic breast cancer. Combination of Doxorubicin and TβRI-KI enhanced the efficacy of doxorubicin in reducing tumor growth and lung metastasis in the 4T1 orthotopic xenograft model in comparison to single treatments. Doxorubicin treatment alone enhanced metastasis to lung in the human breast cancer MDA-MB-231 orthotopic xenograft model and metastasis to bone in the 4T1 orthotopic xenograft model, which was significantly blocked when TβR1-KI was administered in combination with doxorubicin.

**Conclusions:**

These observations suggest that the adverse activation of TGFβ pathway by chemotherapeutics in the cancer cells together with elevated TGFβ levels in tumor microenvironment may lead to EMT and generation of cancer stem cells resulting in the resistance to the chemotherapy. Our results indicate that the combination treatment of doxorubicin with a TGFβ inhibitor has the potential to reduce the dose and consequently the toxic side-effects of doxorubicin, and improve its efficacy in the inhibition of breast cancer growth and metastasis.

## Introduction

Breast cancer is the leading cause of cancer death in women with more than a million newly diagnosed cases annually worldwide [1]. It is estimated that 30–75% of patients undergoing surgery and adjuvant treatment will develop recurrent metastatic disease. Metastatic breast cancer (MBC) is essentially incurable with standard therapy and patients with MBC have a median survival of about 2 years after metastasis have been detected [2]. Doxorubicin is an anthracycline drug widely used in chemotherapy regimen for patients with MBC [3] and shown overall response rates of between 35 and 50% in patients with MBC who have not previously received chemotherapy [4]. Despite its excellent anti-tumor activity, doxorubicin has a relatively low therapeutic index and its clinical utility is limited due to acute and chronic toxicities such as myelosuppression, immunosuppression and dose-cumulative cardiotoxicity [5]. Therefore, combination treatment with another highly effective novel non-toxic drug which can lower the dose of chemotherapeutic agents would be desirable.

Transforming growth factor beta (TGFβ) has been shown to be overly produced during progression of various types of carcinomas including breast cancer [6], [7] and to accelerate metastatic progression [8]–[10]. Several mechanisms are believed to mediate TGFβ's tumor-promoting activity. TGFβ produced by tumor cells can act in a paracrine fashion to stimulate myofibroblast differentiation [11] and tumor angiogenesis [12], and to suppress host immune surveillance [13]. Acting in an autocrine fashion, TGFβ signaling has been shown to be necessary for the survival of breast cancer cells [14], [15] and to induce epithelial-mesenchymal transition (EMT) and cell migration [16]. Due to its oncogenic role, various components of TGFβ pathway are being evaluated as therapeutic targets [17]–[19]. TGFβ type I receptor (TβRI) kinase is one potential target for the blockade of TGFβ signaling [20]. Several studies showed that treatment with TβRI kinase inhibitors (TβRI-KI) can inhibit malignant properties of cancer cells in vitro and in vivo [21]–[25].

Recent studies have demonstrated that EMT induced by TGFβ and other factors is associated with the expression of many stem cell markers and phenotypes in transformed human mammary epithelial cells [26], [27]. These studies suggest that TGFβ-induced EMT may result in the maintenance and formation of stem-like breast cancer cells. This notion is consistent with a recent report demonstrating an enhanced TGFβ isoform expression and pathway activity in CD44+ breast cancer cells [28]. Although TGFβ-associated drug resistance has been described previously [29], these recent findings would suggest that TGFβ-induced drug resistance may be in a large part due to its induction of stem-like features in carcinoma cells. Thus, TGFβ inhibitors should potentiate the efficacy of chemotherapeutic agents, especially for those agents that can adversely activate TGFβ pathway.

It was found that various anti-tumor drugs induced TGFβ1 production in tumor cells [29], [30], which could increase the potential of recurrent disease. In our study, we observed that chemotherapeutic drug doxorubicin activates TGFβ signaling in human breast cancer MDA-MB-231 and murine breast cancer 4T1 cells. Doxorubicin induced EMT, promoted invasion and enhanced generation of cells with stem cell phenotype in murine 4T1 breast cancer cell line in vitro, which were significantly inhibited by TβRI-KI. Our in vivo studies showed for the first time the benefit of simultaneous treatment of doxorubicin with a TGFβ antagonist to improve the inhibition of tumor growth and lung/bone metastasis in a syngeneic immunocompetent murine xenograft model and in a nude mice xenograft model of human breast cancer.

## Results

### Doxorubicin stimulated both motility and invasion of murine and human breast cancer cells

**Figure 1:**
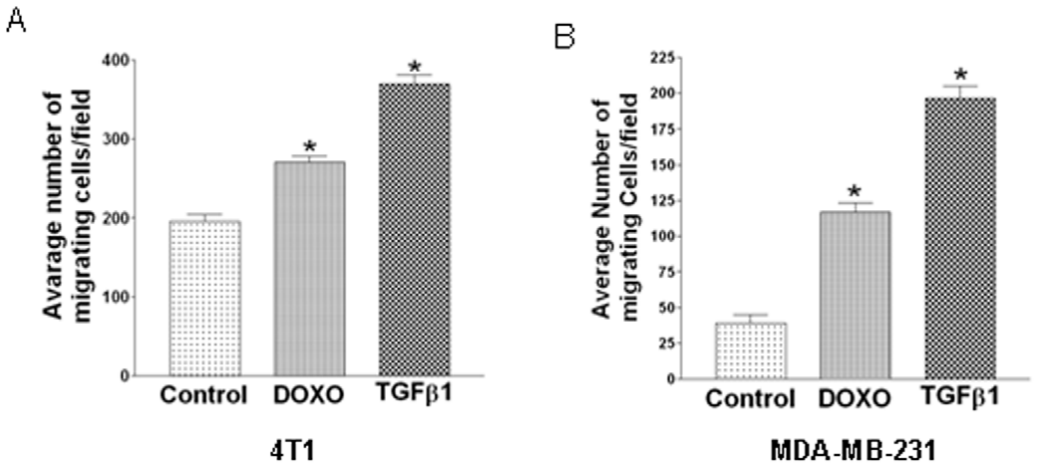
Stimulation of migration by doxorubicin and TGFβ treatment in murine and human breast cancer cells. In a Boyden Chamber migration assay, 40,000 murine breast cancer 4T1 cells (**A**) and human breast cancer MDA-MB-231 cells (**B**) in serum free medium were placed inside the insert of an invasive chamber and treated with doxorubicin (100 nM) or TGFβ1 (5 ng/ml in **A** and 1 ng/ml in **B**) for 6 hours (4T1 cells) and 24 hours (MDA-MB-231 cells). Lower chamber contained the medium with 1% serum as chemoattractant. Cells were removed from the upper surface of the chamber membrane and the membrane was then stained for the migratory cells, which were counted under a microscope. Results are the mean of the five fields of observation at 100× magnification and expressed as mean+SEM. “*” indicates significant difference from the control value with ANOVA test, P<0.05.

Cell motility and invasion are integral processes in the metastatic dissemination of tumor cells. Doxorubicin has been used for the treatment of metastatic breast cancer. Therefore, we tested the effect of doxorubicin on the migration and invasion of murine 4T1 and human MDA-MB-231 cells in a Boyden chamber assay. Surprisingly, instead of inhibition, treatment with doxorubicin significantly enhanced both 4T1 and MDA-MB-231 tumor cell migration (Figure 1A and Figure 1B). Enhanced invasion through matrigel was also observed with both 4T1 and MDA-MB-231 cells after treatment with doxorubicin (Figure S1A and Figure S1B respectively). As expected, TGFβ1 treatment significantly induced both motility and invasion in both cell lines under similar conditions. These results indicated that doxorubicin could act like TGFβ and might have the potential to promote tumor metastasis by stimulating tumor cell motility and invasion.

### Doxorubicin enhanced TGFβ signaling in murine and human breast cancer cells

**Figure 2:**
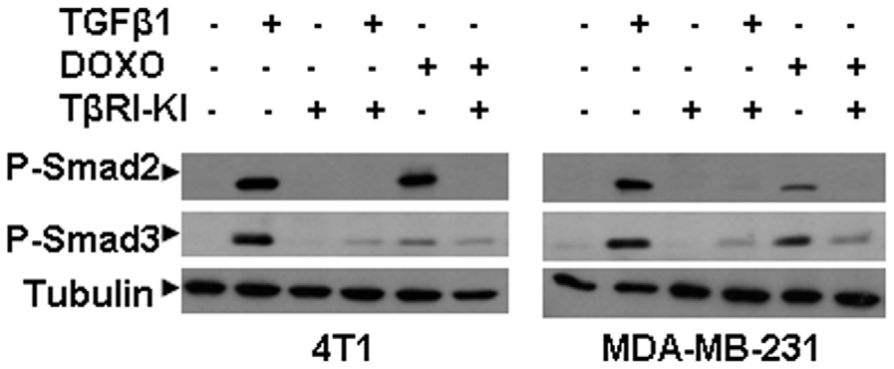
Enhanced phosphorylation of Smad2 and Smad3 in murine and human breast cancer cells by doxorubicin and TGFβ treatment. The murine 4T1 and human MDA-MB-231 breast cancer cells were treated with doxorubicin (25 nM) or TGFβ1(2 ng/ml) in the presence or absence of TGFβ antagonist TbRI-KI (500 nM) for 7 days. The medium was changed every alternate day. The cell extracts were used for Western blotting analysis to measure the levels of phosphorylated Smad2 (P-Smad2) and phosphorylated Smad3 (P-Smad3) with their respective antibodies.

Doxorubicin has been shown to induce circulating TGFβ and lung metastasis in xenograft and transgenic animal models [31], [32]. To examine whether activation of TGFβ signaling has any role in the doxorubicin induced migration/invasion, we examined the effect of doxorubicin treatment on TGFβ-mediated phosphorylation of Smad2 and Smad3 in both murine 4T1 and human MDA-MB-231 cells. After treatment for 6 days, we found that similar to TGFβ1 treatment (2 ng/ml), doxorubicin treatment (25 nM) also stimulated phosphorylation of Smad2 and Smad3 proteins in both 4T1 and MDA-MB-231 (Figure 2) cells, which was inhibited by the addition of the TGFβ inhibitor TβRI-KI. Similar results were also obtained during a shorter duration of treatments (24 hours) with TGFβ3 or doxorubicin in the presence or absence of TβRI-KI (Figure S2A). Like TGFβ1, doxorubicin could also induce the transcriptional activation of a TGFβ-responsive PAI-1 promoter in a dose-dependent manner (Figure S2B). Thus, our results indicate that doxorubicin can activate the TGFβ pathway, which can be antagonized by a kinase inhibitor of TβRI in both breast cancer cell lines in vitro.

### TβRI-KI reversed Doxorubicin- or TGFβ-induced EMT

**Figure 3:**
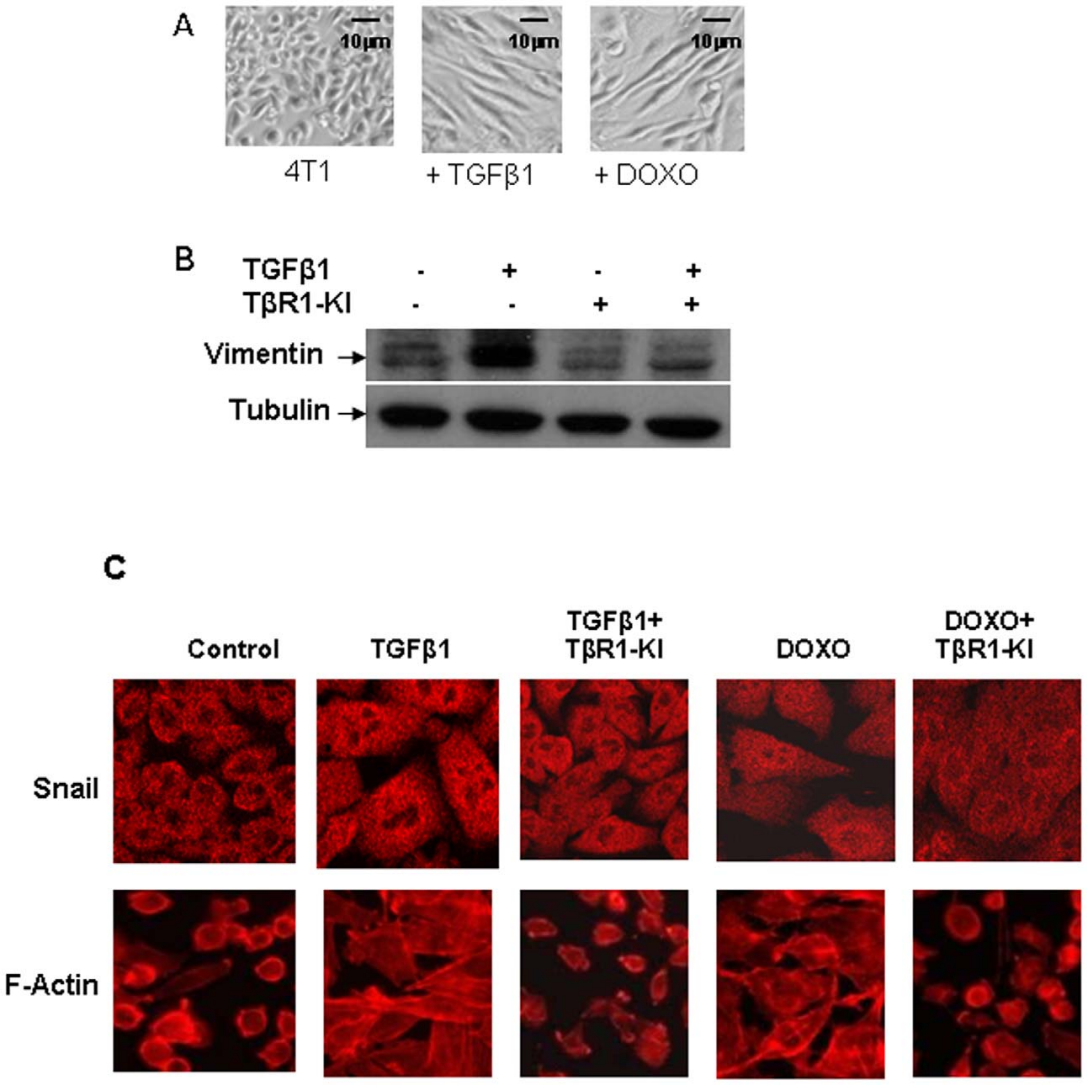
Inhibition of Doxorubicin- and TGFβ-induced EMT by TβRI-KI in murine 4T1 cells. **A.** Representative pictures of 4T1 cells with mesenchymal morphology after treatment with TGFβ1 (2 ng/ml) or doxorubicin (25 nM) for 7 days (×100 magnification). **B.** 4T1 cells were treated with or without TGFβ1 (2 ng/ml) or TbRI-KI (500 nM) for 7 days. Cell lysates were analyzed by western blotting with antibody to Vimentin and tubulin. **C.** 4T1 cells were treated with TGFβ1 (2 ng/ml) or doxorubicin (25 nM) in the presence or absence of TbRI-KI (500 nM) for 7 days. The treated and non-treated 4T1 cells were harvested and about 4×10^3^ cells were seeded on a 8-well Lab-Tek chambered coverglass. After 24 hours, the cells were stained with Rhodamine phalloidin for F-actin or an antibody to Snail. Fluorescent images were taken with a confocal microscope.

Acquisition of mesenchymal phenotype through EMT has been shown to be associated with aggressive breast cancer subtypes and poor clinical outcome in breast cancer patients [33]. TGFβ is a potent inducer of EMT. It induces EMT not only during embryonic development but also during tumor progression in vivo [34]. An essential role for TGFβ mediated Smad signaling has been demonstrated in EMT associated with tumor metastasis [35]. Therefore, we investigated whether the TGFβ- or doxorubicin-induced EMT phenotype can be reversed by the treatment with the TβRI-KI. Due to the spindle shaped morphology of the human breast cancer MBA-MB-231 cells, the detection of additional EMT was difficult. F-actin staining of the cytoskeleton could detect a small number of MDA-MB-231 cells with an enhanced mesenchymal phenotype which was abrogated by the addition of TβRI-KI (Figure S3A and S3B). To study the effect of TβRI-KI on the EMT generated by doxorubicin, therefore we have focused on murine 4T1 cells with an epithelial morphology. The treatment of murine 4T1 cells with TGFβ1 (2 ng/ml) and doxorubicin (25 nM) for 7 days generated majority of the cells with mesenchymal spindle-shaped morphology (Figure 3A). Vimentin, which is a marker associated with mesenchymal morphology, showed increased expression after TGFβ1 treatment and was significantly reduced by the addition of TβRI-KI (Figure 3B). However, the level of vimentin expression was not increased after doxorubicin treatment (data not shown) suggesting that doxorubicin may activate other pathways in addition to the TGFβ pathway to regulate vimentin expression. EMT was evident after treatment with TGFβ1 or doxorubicin as detected by polarized organization of the actin cytoskeleton (Figure 3C). We also observed an increased nuclear translocation of the transcription factor Snail, which is known to promote EMT [36] after the treatment of the 4T1 cells with TGFβ1 or doxorubicin (Figure 3C). The cells regained their epithelial phenotype when TβRI-KI was included in the treatment, suggesting the inhibition of EMT as indicated by the re-expression of F-actin along cell membrane and a more even distribution of Snail between cytoplasm and nucleus (Figure 3C). Our results indicated that TβRI-KI has the potential to reverse the EMT process by counteracting the action of TGFβ and doxorubicin in inducing EMT in murine breast cancer cells in vitro.

### Doxorubicin or TGFβ treatment generated stem cell phenotype with drug resistance

**Figure 4:**
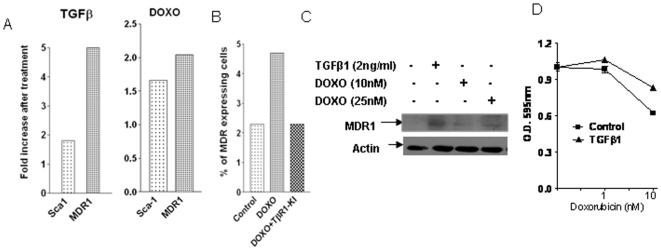
Doxorubicin and TGFβ treatment generated drug resistant cells with a stem cell phenotype. **A** and **B.** The murine 4T1 breast cancer cells were treated with TGFβ1 (2 ng/ml) or doxorubicin (25 nM) in the presence or absence of TbRI-KI (500 nM) for 7 days. The medium was changed every alternate day. The treated and non-treated cells were harvested with trypsinization and incubated with an alexafluor 405-conjugated antibody (2 µg/10^6^ cells) against MDR1 or a Phycoerythrin-conjugated antibody against Sca-1. Fluorescence Activated Cell Sorting (FACS) analysis was carried out using FACSAria flow cytometer (Becton Dickinson). Results were expressed as fold increase of the number of cells expressing the surface markers for Sca-1 or MDR1 after treatment with TGFβ1 or doxorubicin compared to the untreated cells (**A**) or as the percentage of MDR1 expressing cells after doxorubicin treatment in the presence or absence of TβR1-KI (**B**). **C.** 4T1 cells were treated with or without 2 ng/ml TGFβ1 and DOXO (10 nM and 25 nM) for 7 days and cell extracts were used for Western blot analysis with antibody to MDR1. **D.** 4T1 cells, pre-treated with TGFβ1 (5 ng/ml) for seven days, were plated in a 96-well plate and were treated with doxorubicin at two different concentrations (1 nM and 10 nM) in the presence or absence of TGFβ1 (5 ng/ml). Relative cell number after 5 days of incubation was obtained by MTT assay. Each data point is the mean±SEM from 4 replicate wells.

Recently, EMT has been implicated in the generation of stem like cells in normal and transformed human mammary epithelial cells [26], [27]. The authors in both studies showed that prolonged treatment of cells with TGFβ1 enhanced expression of markers associated with EMT and many properties of stem cells which include expression of cell surface stem cell markers and ability to form mammospheres. We examined whether murine breast cancer 4T1 cells, which showed EMT upon treatment with TGFβ1 or doxorubicin, could exhibit stem cell characteristics in vitro. After 7 days of treatment with either doxorubicin or TGFβ1, the cells were analyzed by flow cytometry for the murine stem/progenitor cell marker Sca-1 and the multidrug resistance protein MDR1. A Sca-1 positive population in murine mammary gland has been shown to be enriched for functional stem/progenitor cells as demonstrated by its increased regenerative potential in comparison to the Sca-1 negative cells when transplanted into the cleared mammary fat pads of host mice [37]. Multi drug resistance protein has been implicated in cancer stem cell resistance to chemotherapy [38]. In comparison to the non-treated cells, the number of cells expressing Sca-1 and MDR1 were enhanced by about 2-fold and 5-fold respectively after TGFβ1 treatment and by about 1.7-fold and 2-fold respectively after doxorubicin treatment (Figure 4A). The addition of TβR1-KI during doxorubicin treatment reduced the number of MDR1 expressing cells to the basal level (Figure 4B). Doxorubicin treatment showed enhanced MDR1 expression in a dose dependent manner as detected by Western blotting (Figure 4C). Since TGFβ1 treatment increased MDR1 expression in 4T1 cells which was detected by both flow cytometry (Figure 4A) and western blotting (Figure 4C), we also tested whether TGFβ1 treatment can induce doxorubicin resistance with a cell proliferation assay. We found that treatment with TGFβ1 attenuated growth inhibitory activity of doxorubicin (Figure 4D), which indicates that the stem cell phenotype induced by TGFβ appears to enable breast cancer cells to acquire resistance to anti-cancer agents like doxorubicin. Thus, our results indicated that doxorubicin or TGFβ1 treatment has the potential to generate drug resistant cancer cells with stem/progenitor cell features.

### TβRI-KI reduced doxorubicin-induced mammosphere formation

**Figure 5:**
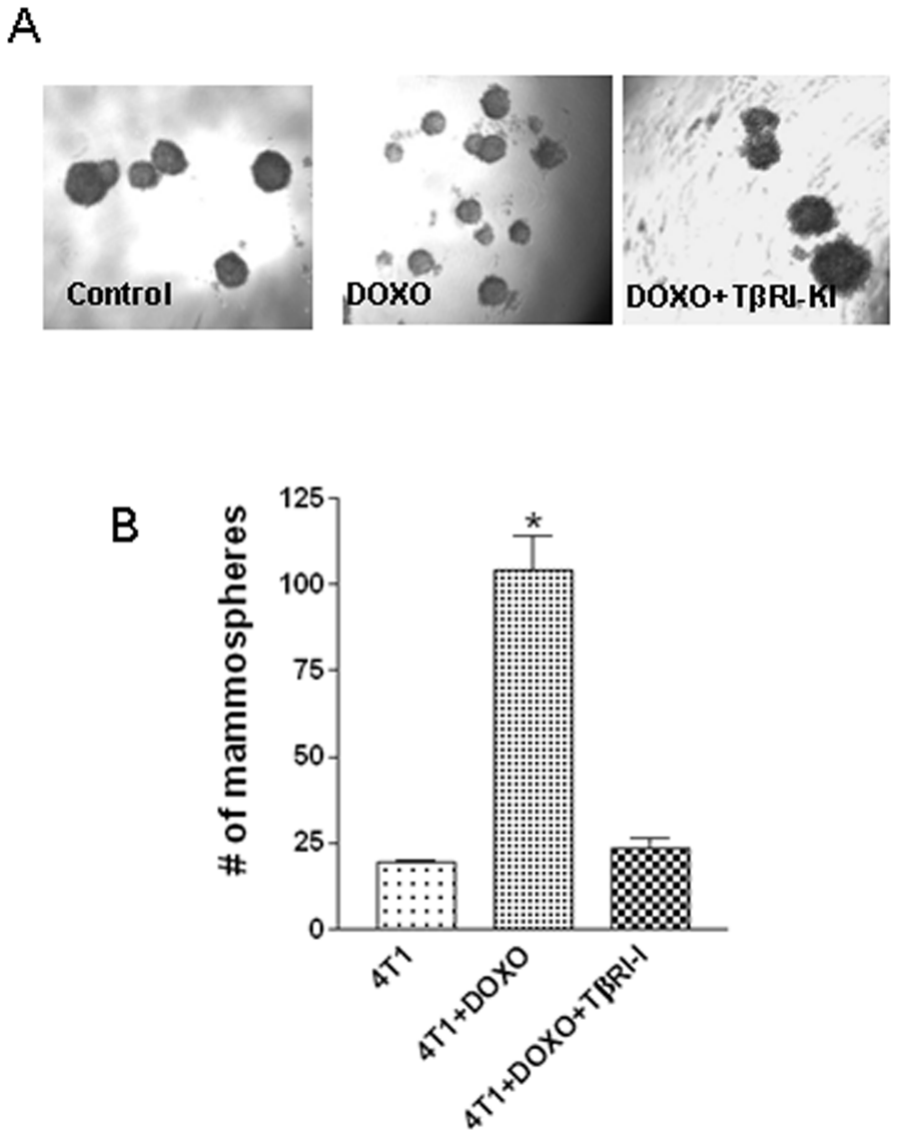
Abrogation of doxorubicin induced mammosphere formation by TβRI-KI. Murine 4T1 cells were treated with doxorubicin (25 nM) in the presence or absence of TbRI-KI for 6 days. The cells with or without treatment were harvested after trypsinization and filtered through 40 µm strainer to obtain a single-cell suspension. Cells were seeded (1,000 cells/100 µl/well) in a 96-well ultra-low attachment plate (Costar) and mammospheres were counted after 7 days of incubation. **A.** Representative pictures of mammospheres obtained from cells with or without doxorubicin treatment and also after doxorubicin treatment in the presence of TbRI-KI. **B.** The number of mammospheres obtained from 1,000 cells in triplicate wells with or without treatments were counted and expressed as mean±SEM. “*” indicates significant difference from the control value, P<0.05.

One defining characteristic of the stemness of a cell is its ability to self-renew and to generate differentiated progeny. Recently, a non-adherent and non-differentiating culture system has been developed [39] in which a small population of stem like mammary epithelial cells, capable of surviving anoikis and proliferating in such conditions, formed discrete clusters of cells termed ‘mammospheres’. Each of these spheroid represents a mammary stem/early progenitor cell which undergoes limited self-renewal and then gives rise to mammary progenitors capable of multi-lineage differentiation. It has been shown that mammosphere cultures from tumor cell lines and human breast metastasis specimens enrich for tumor initiating cells [40]. Induction of EMT has been shown to promote mammosphere formation in primary human mammary epithelial cells after prolonged treatment with TGFβ [26]. We also observed that treatment of 4T1 cells with TGFβ1 (0.05 and 0.1 ng/ml) for 12 days stimulated the formation of mammospheres (Figure S4). We have used this model to study whether doxorubicin induced EMT can promote the formation of mammospheres which represents self-renewing stem/early progenitor cells. We found a 5-fold increase in the number of mammospheres formed by the treatment of 4T1 cells with 25 nM doxorubicin for 7 days, in comparison to the mammospheres formed by the non-treated cells. This enhanced formation of mammospheres by doxorubicin was drastically reduced by the addition of TβR1-KI (Figure 5A and Figure 5B).

Thus, our in vitro results revealed that the chemotherapeutic agent doxorubicin can induce TGFβ signaling, promote EMT and generate cells with drug resistant stem cell phenotypes in murine breast cancer 4T1 cells, which could be reversed by the treatment with a TGFβ antagonist TβRI-KI. These results prompted us to test the hypothesis that antagonism of increased TGFβ signaling during doxorubicin treatment in vivo may enhance anti-tumor activity of doxorubicin in the murine breast cancer 4T1 and human breast cancer MDA-MB-231 xenograft models.

### TβRI-KI enhanced the efficacy of doxorubicin to reduce tumor growth and spontaneous lung metastasis by murine breast cancer cells

**Figure 6:**
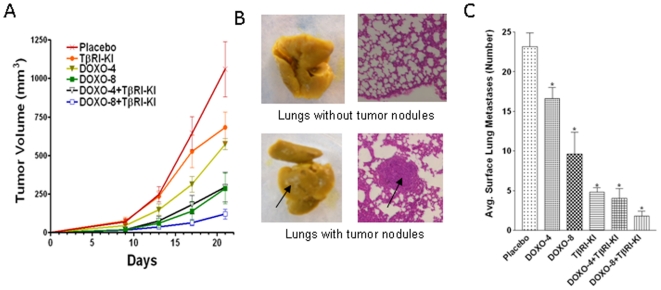
TGFβ inhibitor enhances the efficacy of doxorubicin treatment to reduce tumor growth and spontaneous lung metastasis of murine breast cancer cells. **A.** Murine breast cancer 4T1 cells (0.1×10^6^) were inoculated at the both inguinal mammary fat pad of syngeneic Balb/c mice and divided into six groups. Animals were treated with PBS (Placebo), TβRI-KI at 1 mg/kg every alternative day, doxorubicin (DOXO) at 4 mg/kg (DOXO-4) or 8 mg/kg (DOXO-8) once per 7 days, or the combination of TβRI-KI and DOXO-4 or DOXO-8 through i.v. injection starting at the day of tumor cell inoculation. Tumor sizes were measured twice weekly with a caliper, and the tumor volumes were calculated with the equation V = (L×W^2^)×0.5, where L is length and W is width of a tumor. Experiment was terminated after 3 wks of treatment and the final tumor volume (sum of two tumors per mouse) was used for calculating mean tumor volume ± SEM from 6 mice in Placebo and 5 mice in the rest of the groups. **B and C.** After the termination of the experiment, lungs were excised and fixed in Bouin's solution (Sigma) to count surface lung metastasis, followed by paraffin embedding for histological H & E staining. Representative picture of lungs without and with metastatic nodules are shown with corresponding histological identification of the nodules by H & E staining (**B**). The visible surface lung nodules were counted. “*” denotes a significant difference (P<0.005) from the corresponding control with a Student *t* test (**C**).

Balb/c mice were injected with 4T1 cells at both inguinal mammary glands and treated immediately once a week with a low or a high dose of doxorubicin (4 mg and 8 mg/kg body weight), or with TβRI-KI (1 mg/kg body weight) every alternate day, either alone or in combination, by i.p. injection. Treatment with TβRI-KI for 21 days moderately inhibited primary tumor growth of 4T1 tumors while treatment with doxorubicin induced a dose-dependent and more noticeable inhibition in comparison to the placebo treatment (Figure 6A). However, much more significant inhibition of tumor growth was obtained when the treatment of TβRI-KI was combined with doxorubicin at both low (DOXO-4) and high doses (DOXO-8) (Figure 6A). In fact, the combination of TβRI-KI and the low dose of doxorubicin produced a similar inhibitory effect to that of the high dose of doxorubicin alone. After the termination of the experiment, metastatic tumor nodules on the surface of the lungs, as shown in Figure 6B, were counted. The metastatic burden on the lungs, as represented by the number of tumor nodules, was moderately, but dose dependently, lowered by doxorubicin treatments. Interestingly, while TβRI-KI was not as effective as doxorubicin in inhibiting orthotopic tumor growth, it was much more effective in blocking lung metastasis than doxorubicin suggesting that metastasis can be independent of primary tumor size (Figure 6C). The combination between TβRI-KI and the higher dose of doxorubicin produced greater inhibition of lung metastasis than either treatment alone (Figure 6C). Thus, combination treatment of doxorubicin and TβRI-KI effectively enhanced the inhibition of both tumor growth and spontaneous lung metastasis of murine 4T1 cells in comparison to the single treatments.

### TβRI-KI inhibited doxorubicin-induced spontaneous lung metastasis of human breast cancer cells

**Figure 7:**
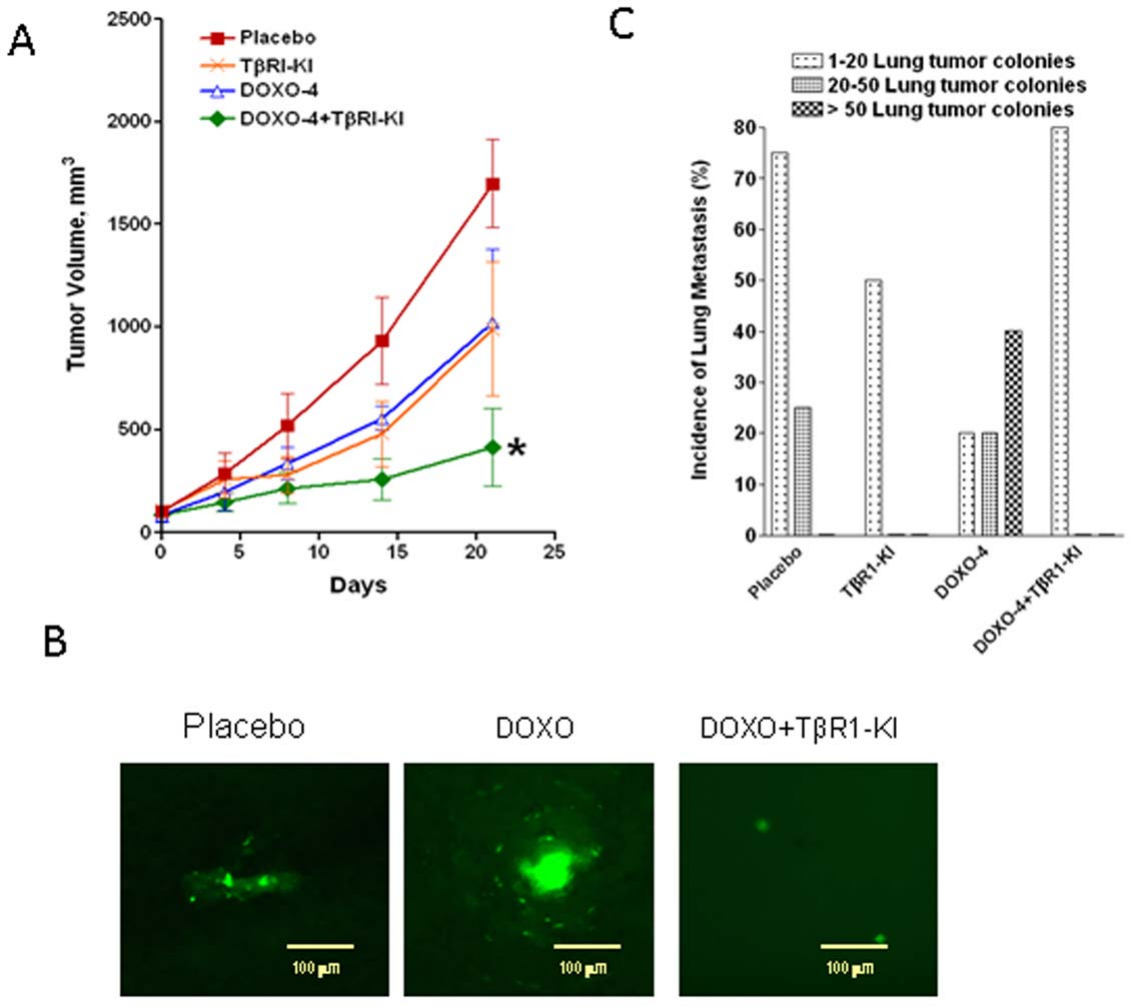
Treatment with TβRI-KI blocked doxorubicin-induced spontaneous lung metastasis of human breast cancer cells. **A.** EGFP-expressing sub confluent MDA-MB-231 cells (1×10^6^) were inoculated into both inguinal mammary fat pads of female athymic nude mice. Tumor volume was measured externally and calculated as described in the legend of Figure 5. Tumors were allowed to reach 80–100 mm^3^ in size and the animals were then ranked according to their tumor burden and divided into four groups such that the mean and the median of the tumor volume of the groups are closely matched. Animals were treated with PBS (control), TβRI-KI every alternative day, doxorubicin once every week, and with the combination of TβRI-KI and doxorubicin respectively. The experiment was terminated after 3 weeks and the final tumor volume (sum of two tumors per mouse) was used for calculating mean tumor volume±SEM from 4 mice in control and TβRI-KI groups and 5 mice in the other two groups. Asterisk “*” denotes significant difference (P<0.05) of the terminal tumor volumes between control and the combination treatment group by ANOVA. **B.** Representative Pictures of lung metastasis colonies formed by EGFP-expressing MDA-MB-231 cells detected by fluorescence microscopy (×200 magnification). **C.** Green fluorescent micrometastatic colonies in the whole lung were detected and counted. The percent of mice with lung metastasis in each group is expressed as total lung metastasis incidence. Incidence as percent of mice with numbers of lung metastasis colonies ranging between 1 and 20, between 20 and 50, and >50 is also presented.

We also utilized the human breast cancer MDA-MB-231 cells expressing enhanced green fluorescence protein (EGFP) to investigate the effects of doxorubicin and/or the TGFβ antagonist TβRI-KI treatment on metastasis induced by orthotopic xenograft tumors formed by the MDA-MB-231 cells. The treatment was initiated when the average tumor volume was between 80–100 mm^3^. After 21 days of treatment with both i.v. injection of doxorubicin (4 mg/kg/wk) and i.p. injection of the TβRI-KI (1 mg/kg/2days), either alone or in combination, we examined the tumor growth rate and the spontaneous lung metastasis. Similar to the 4T1 tumor model, combination treatment significantly reduced tumor growth in comparison to the single treatments although treatment was started after the initiation of tumor growth (Figure 7A). Most interestingly, examination of the whole lungs revealed that the size of the metastatic colonies was much larger in doxorubicin group than in the control group as observed by EGFP fluorescence imaging (Figure 7B). Furthermore, there was increased number of mice with greater than 50 lung metastatic colonies in the doxorubicin-treated group in comparison to the control group (Figure 7C) even though doxorubicin treatment moderately reduced primary tumor growth (Figure 7A) indicating that doxorubicin alone can stimulate metastasis. Treatment with TβRI-KI also moderately inhibited tumor growth, however a statistically significant reduction of tumor volume was only observed with the treatment of both doxorubicin and TβRI-KI in comparison to that of the control group (Figure 7A). More significantly, the combination treatment remarkably inhibited lung metastasis as evidenced by reduced lung metastatic colonies both in colony size (Figure 7B) and number (Figure 7C′). The size of lung metastatic colonies in TβRI-KI group was similar to that in the combination treatment group (picture not shown). The fact that the number of mice with 1–20 lung metastases in the combination treatment group was still higher than that in TβRI-KI alone group suggests that TβRI-KI treatment did not completely inhibit doxorubicin-induced lung metastasis. Thus, our study indicates that the TGFβ inhibitor has the potential to inhibit doxorubicin-induced lung metastasis.

### TβRI-KI reduced doxorubicin-induced spontaneous bone metastasis in a murine xenograft model

**Figure 8:**
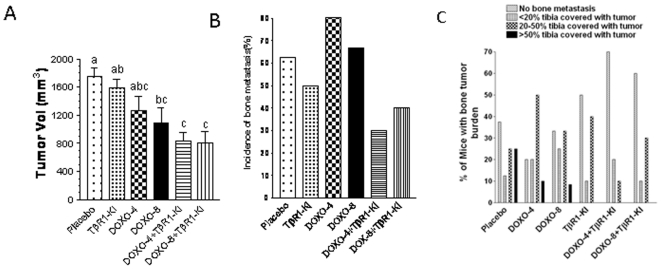
TβRI-KI reduced doxorubicin-induced spontaneous bone metastasis in the murine xenograft model. **A.** 4T1 cells (0.1×10^6^) were inoculated into both inguinal mammary fat pads of syngeneic female balb/c mice. When the average tumor volume reached 60–70 mm^3^ after 2 weeks, the mice were divided into six groups with similar tumor burden and treated i.p. with PBS (control), TβRI-KI at 1 mg/kg every alternative day, doxorubicin at 4 mg/kg (DOXO-4) or 8 mg/kg (DOXO-8) once per 7 days, or the combination of TβRI-KI and DOXO-4 or DOXO-8. The experiment was terminated after 3 wks of treatment and the final tumor volume (sum of two tumors per mouse) was used for calculating mean tumor volume±SEM from 6 mice in DOXO-8 and 5 mice in the rest groups. The bars bearing a different letter are significantly different (P<0.05) by ANOVA. **B.** After the termination of the experiment, the leg bones (Femora and tibiae) were fixed in 10% neutral buffered formalin, decalcified in 10% EDTA and embedded in paraffin. Sections were stained with H&E, orange G and phloxine. The presence of tumors in the bone marrow was examined microscopically. The incidence of bone metastasis in each group of animals was expressed in percentage of the number of animals with bone metastasis. **C.** Animals with bone tumor burden were divided into four groups and the results were expressed in percentage of animals with no bone metastasis, with <20% tibia replaced by tumor, with 20–50% tibia replaced by tumor, and with >50% of tibia replaced by tumor.

Breast cancer metastasizes to multiple sites and frequently to the skeleton, resulting in poor prognosis. In our previous study we found that systemic administration of the TβRI-KI can inhibit bone metastasis of human cancer cells in an intra-cardiac injection model of bone metastasis [22]. Orthotopic tumors formed by 4T1 cells have been shown to induce spontaneous bone metastasis [41]. However, we failed to detect tumor cells in the sections of femora and tibiae from either the control or the treated mice in our experiment described in Figure 6. We suspected that the experiment duration of three weeks might not be long enough for metastatic 4T1 cells to form tumors in bony tissue. Therefore, we performed another set of experiment with the 4T1 cells, in which the treatment with doxorubicin and/or TβRI-KI was delayed until the orthotopic xenograft tumors reached an average size of 60–70 mm^3^. Doxorubicin was administered by i.v. injection and TβRI-KI by i.p. injection for 21 days. This combination treatment inhibited the tumor growth in comparison to the single treatments (Figure 8A). Tibia specimens in all treatment groups were analyzed for the incidence of bone metastasis, tumor burden and osteolysis indicated by the loss of trabecular bone after histological staining. Metastatic 4T1 tumors in different specimens were seen at different areas of tibia, often away from the trabecular bone area. Therefore, osteolysis as reflected by loss of trabecular bone could not be compared between the placebo and the treated mice with bone metastasis. More metastatic tumors in the tibia were seen after single treatment with either dose of doxorubicin than treatment with TβRI-KI, which moderately reduced the incidence of metastasis in comparison to the placebo treatment group (Figure 8B). More significantly, the metastasis incidence was reduced by 50–60% when animals were treated with both doxorubicin and TβRI-KI (Figure 8B). We also compared the percent incidence of different levels of the tibial tumor burden among the treatment groups. We recorded the levels of tumor burden as: no bone metastasis, <20% tibia replaced by tumor, 20–50% tibia replaced by tumor, and >50% of tibia replaced by tumor. The percentage of specimens with higher tumor burden (>50%) was evident only in the placebo or doxorubicin-treated groups but not in the TβRI-KI-treated or the combination treatment groups (Figure 8C). About 70% animals were free of bone metastasis after the combination treatment in comparison to about 24% and 32% in the low and high dose doxorubicin treatment group respectively (Figure 8C). These results demonstrated that doxorubicin treatment alone increased the bone metastasis incidence and bone tumor burden which were remarkably reduced by the simultaneous treatment with a TGFβ inhibitor.

Although the combination treatment after tumors were formed and growing was effective in inhibiting bone metastasis, its effect on lung metastasis was much less obvious because the majority of lungs in each treatment group were covered with metastatic nodules making it difficult to count the nodules. Thus, our results indicate that early treatment with the combination of doxorubicin and TβRI-KI will likely be much more effective than delayed treatment in blocking lung metastasis.

## Discussion

In this study, we have evaluated the potential therapeutic benefit of using a small molecule TGFβ signaling inhibitor with doxorubicin in animal models of breast cancer. Doxorubicin, which is a frequently used chemotherapeutic agent against metastatic breast cancer, has been reported to induce acute and chronic toxicities and immunosuppression. In addition, we found in our study that doxorubicin can activate TGFβ signaling in cancer cells, which has the potential to accelerate malignant tumor progression. Thus, novel treatment options are needed to reduce dosages of doxorubicin and consequently unwanted toxicities while enhancing its therapeutic efficacy in treating metastatic cancer.

An elevated plasma level of TGFβ has been correlated to the progression of various types of malignancies including metastatic breast cancer progression [9] and survival [42]. Persistent high levels of circulating TGFβ predicted early metastatic recurrences in the liver of patients with colorectal cancer after curative resection [43]. In addition to the carcinogenesis-associated upregulation of TGFβ expression, several chemotherapeutic agents including doxorubicin were shown to stimulate TGFβ1 expression and secretion from xenograft tumors [31]. Surprisingly, in a recent study, it was shown that doxorubicin treatment caused increased circulating levels of TGFβ as well as increased circulating tumor cells and lung metastases in the MMTV/PyVmT transgenic animal model [32]. We have observed that the presence of doxorubicin induced the migration and invasion of murine 4T1 and human MDA-MB-231 breast cancer cells similar to the TGFβ1 treated cells in a Boyden chamber assay. Further investigation revealed that doxorubicin stimulated the phosphorylation of Smad2 and Smad3 in both 4T1 and MDA-MB-231 cells. Consistent with the stimulation of Smad2 and Smad3 phosphorylation, doxorubicin also stimulated the TGFβ-responsive PAI-1 promoter activity in a dose dependent manner. Our results strongly indicate that although doxorubicin can induce cytostatic and/or cytocidal effects, it can also induce cancer cell migration/invasion by activating TGFβ pathway.

EMT is a latent embryonic program by which some cells within a tumor during cancer progression can reactivate mesenchymal traits to disseminate to metastatic sites [33]. Induction of EMT by TGFβ has been shown to increase motility and invasiveness via the disassembly of cell-to-cell contacts, loss of cell polarity and significant cytoskeletal reorganization in normal and malignant mammary epithelial cell types. We observed in the murine breast cancer 4T1 cell model that TGFβ and surprisingly also doxorubicin can induce EMT as evidenced by the mesenchymal morphology, reorganization of the actin cytoskeleton and nuclear localization of the embryonic transcription factor Snail.

Emerging evidence has shown that certain type of tumors including breast tumors contain a subset of cells that initiate and propagate the tumors with high efficiency. These cells are referred as cancer stem cells (CSCs) and often exhibit phenotypes resembling normal stem cell properties such as expression of stem cell markers, self renewal and multipotency [44]. Metastatic breast cancer cells are notorious for their ability to survive treatment and regenerate tumors. One important feature of CSCs is their resistance to the cytotoxicity of varous chemotherapeutic agents because of their relatively high expression levels of multiple drug resistance transporters such as ABCB1 (MDR1/PGP, P-glycoprotein) and ABCG2 (MXR/BCRP) [38]. Thus, chemotherapeutics might spare CSC's and allow recurrence of more aggressive tumors. Recent data showed that immortalized human and murine breast cancer cell lines contain stem-like cells similar to CSCs isolated from primary tumor tissue with identical cell surface markers, clonogenic and sphere formation potential, and in vivo functional transplantation properties [45]. The fact that EMT can generate stem-like cancer cells [26] suggests that therapeutic-resistant cancer stem cells may be the result of either the transformation of normal stem cells or via the induction of EMT in more differentiated cancer cells. Indeed, we found that treatment with TGFβ or doxorubicin increased the number of 4T1 cells expressing stem cell marker Sca-1 and the drug resistant transporter protein MDR1, and with the stem cell property of self-renewal as indicated by mammosphere formation. The 4T1 cells pre-treated with TGFβ1 for 7 days showed resistance to the doxorubicin-induced growth inhibition.

Our results indicate that by activating TGFβ signaling, doxorubicin has the ability to induce EMT and generate tumor cells with stem cell phenotypes. The EMT program is often considered a transient and reversible process. Studies of embryonic development have showed that mesenchymal cells of mesoderm can give rise to epithelial organs, including the kidney and ovary, by undergoing the reverse EMT [46]. The finding that EMT plays a role in the development of cancer stem cells suggests that therapies blocking or reversing the EMT program may sensitize resistant cancer stem cells to conventional cancer therapies. We have evaluated the ability of a small molecule TGFβ inhibitor, TβRI-KI, to block the EMT process and subsequent cancer stem cell formation by TGFβ1 and doxorubicin treatment in 4T1 cells in vitro. We found that blockade of TGFβ signaling significantly reversed the TGFβ1- and doxorubicin-mediated EMT process. Restoration of the epithelial phenotypes of the cytoskeleton organization and cytoplasmic localization of the EMT transcription factor Snail was observed in the presence of the inhibitor. The treatment of TβRI-KI also inhibited TGFβ1- and doxorubicin-mediated generation of cells with stem cell phenotypes including the expression of MDR1 and the formation of mammospheres.

Thus, all our in vitro experiments indicated potential therapeutic benefits of using TβRI-KI to antagonize the doxorubicin-induced TGFβ signaling, which has the potential to induce EMT and to generate drug resistant stem-like cancer cells. We designed in vivo experiments utilizing murine breast cancer 4T1 cells and human breast cancer MDA-MB-231 cells to study the synergistic anti-tumor effect of the systemic administration of both doxorubicin and TβRI-KI in comparison to single-agent treatments. Our studies revealed that while treatment with either agent alone moderately inhibited tumor growth in both xenograft breast cancer models, the combination of both agents was clearly much more effective in inhibiting tumor growth. In fact, a 50% reduction of doxorubicin dose was sufficient to produce the same tumor growth inhibition as the full dose when TβRI-KI was added to the treatment suggesting that the combination with TGFβ antagonists may be an effective strategy in reducing doxorubicin dosage and its unwanted side-effects.

Lung metastasis is prevalent among the patients with metastatic breast cancer [47]. About 60% to 74% of patients who die of breast carcinoma have lung metastasis [48]. In several animal models, TGFβ has been shown to promote lung metastasis [49], [50]. Since Doxorubicin is widely used as an adjuvant chemotherapeutic agent in metastatic breast cancer with limited response rate (4) and enhanced lung metastasis in an animal model [32], we also examined the lungs of the animals for the presence of metastatic colonies in the placebo and treatment groups. We observed moderate reduction of lung metastasis after doxorubicin treatment which was significantly improved by combinational therapy with TβRI-KI in the murine 4T1 xenograft model. In the human breast cancer xenograft model, our results indicated that doxorubicin alone can stimulate lung metastasis with larger metastatic colonies which can be effectively blocked by the simultaneous treatment with TβRI-KI. Since smaller sized pulmonary metastasis has been shown to be significantly associated with longer survival [47], our results suggest that administration of TβRI-KI along with doxorubicin may have the potential to improve the survival rate of doxorubicin-treated metastatic breast cancer patients.

Metastases to bone are frequently seen pathological lesions in advanced breast cancer, which causes debilitating skeletal complications such as osteolysis, intractable bone pain and pathologic fractures [51]. Most importantly, once breast tumor cells metastasize to bone marrow, mortality increases to 70% in comparison to 40% in the breast cancer patients with no tumor cells in their bone marrow [52], [53]. Therefore, prevention and treatment of bone metastasis is important for the improvement of survival in breast cancer patients. In our study, we observed that mice bearing orthotopic 4T1 tumors showed an increased incidence of spontaneous bone metastasis as well as greater bone tumor burden after doxorubicin treatment in comparison to the placebo treatment. Both incidence of tumor cells and tumor volume in the bone marrow were reduced remarkably after simultaneous treatment with TβRI-KI even though TβRI-KI treatment alone was only moderately effective in reducing bone metastasis incidence and tumor burden. Thus, the combination therapy appears to produce a synergistic inhibition of bone metastasis in this model system.

In summary, our study suggests that doxorubicin at certain dosages can promote breast cancer invasion and metastasis in part through its ability to activate TGFβ signaling, which in turn generates stem-like cancer cells with increased drug resistance through an EMT process. Our study also suggests that administration of TGFβ antagonists during chemotherapy may sensitize carcinoma cells, in part through the inhibition of EMT, to the anti-tumor and anti-metastatic activities of chemotherapy resulting in increased therapeutic efficacy and reduced dosage of chemotherapeutics and their unwanted side effects.

## Materials and Methods

### Ethics statement

All animal experiments were conducted following appropriate guidelines. They were approved by the Institutional Animal Care and Use Committee and monitored by the Department of Laboratory Animal Resources at the University of Texas Health Science Center at San Antonio.

### Cell lines

Human breast cancer cell line MDA-MB-231 and murine breast cancer 4T1 cell line was originally obtained from the American Type Tissue Culture Collection (ATCC). These cell lines were cultured in McCoy's 5A medium supplemented with pyruvate, vitamins, amino acids, antibiotics, and 10% fetal bovine serum (FBS) as described previously [54].

### Western Blotting

The murine 4T1 and human MDA-MB-231 breast cancer cells were treated with doxorubicin (25 nM) in the presence or absence of TGFβ1 (2 ng/ml) and/or TβRI-KI (500 nM) for 7 days. The medium was changed every alternate day with the addition of doxorubicin and TGFβ1 each time. The cell lysates were used in the western blotting analysis as described previously [14]. Antibody to the Phospho-Smad2 was from Cell signaling, to vimentin was from Sigma, and to Phospho-Smad3 and MDR1 was from Santa Cruz Biotechnology. The TβRI-KI used in our study was previously reported to be an ATP competitive inhibitor of the TGFβ RI kinase [55], [56]. The compound, [3-(pyridine-2yl)-4-(4-quinonyl)]-1H pyrazole, was synthesized according to the procedure described by Sawyer et al. [55].

### In vitro cell migration and invasion assays

Cell migration and invasion assays were performed in 24-well Boyden Chambers with 8-µm pore polycarbonate membranes (Becton Dickinson Labware, Rockville, MD) for migration assays and with matrigel coated membranes for invasion assay. For migration assay, 40,000 murine breast cancer 4T1 cells and human breast cancer MDA-MB-231 cells in serum free medium were seeded in the upper chamber and treated with doxorubicin (100 nM) or TGFβ1 (5 ng/ml) and incubated for 6 hours and 24 hours respectively. Lower chamber contained the medium with 1% serum as chemoattractant. After incubation, cells were removed from the upper surface of the chamber membrane with a cotton swab and the membrane was then stained for the migratory cells at the lower surface of the chamber with Hema 3 stain kit (Fisher Scientific, Houston, TX) according to the manufacturer's protocol. The stained cells were counted under a microscope. For invasion assay using matrigel coated chambers, 50,000 murine 4T1 cells and human MDA-MB-231 cells in serum free medium were used and treated similarly as described above with doxorubicin or TGFβ1 for 18 hours and 24 hours respectively. Lower chamber contained the complete medium with 10% serum as chemoattractant. Invasive cells were stained and counted as described above.

### Mammosphere culture

Murine 4T1 cells were treated with doxorubicin (25 nM) for 6 days in the presence or absence of TβRI-KI (500 nM). The medium with doxorubicin was changed every alternate day. The cells with and without treatment were harvested after trypsinization with 0.05% trypsin and filtered through a 40-µm strainer to obtain a single-cell suspension. Cells were seeded (1000 cells/100 ul/well) in a 96 well ultra-low attachment plate (Costar) in DMEM-F12 medium supplemented with 2% B27 (Gibco), which is a serum replacement that excludes constituents that lead to cell differentiation and 20 ng/ml EGF, 20 ng/ml bFGF, 0.5 mg/ml hydrocortisone, 5 mg/ml insulin and gentamycin/amphotericin-B (Lonza). Mammospheres were counted after 5–7 days of incubation.

### Flow Cytometry

The murine 4T1 breast cancer cells were treated with doxorubicin (25 nM) in the presence or absence of TGFβ1 (2 ng/ml) and/or TβRI-KI (500 nM) for 7 days. The medium was changed every alternate day with the addition of doxorubicin and TGFβ1 each time. The harvested treated and non-treated cells after trypsinization were stained for flow cytometry at a concentration of 1×10^6^ cells per 100 µl of buffer (PBS pH 7.4, 2% PBS, 2 mM EDTA). Cells were incubated with conjugated antibodies (2 µg/10^6^ cells) against MDR (Anti MDR-alexafluor 405, Santa Cruz Biotechnology) and Sca-1 (anti Sca-1-PE, BD Biosciences) at 4°C for 20 min. Fluorescence activated cell sorting (FACS) analysis was carried out using FACSAria flow cytometer (Becton Dickinson) at the core imaging facility of the UT Health Science Center at San Antonio, Texas.

### Cell proliferation assay

4T1 cells pre-treated with TGFβ1 for seven days were plated in a 96-well plate at 2,000 cells/well. Cells were treated with doxorubicin at two different concentrations (1 nM and 10 nM) in the presence or absence of TGFβ1 (5 ng/ml) and 3-(4,5-dimethylthiazol-2-yl)-2,5-diphenyltetrazolium bromide (MTT) was utilized to obtain the relative cell number after 5 days of incubation as described previously [22].

### Immunofluorescence

The treated and non-treated cells (as described earlier) were harvested and about 4×10^3^ cells were seeded on a 8-well Lab-Tek chambered coverglass (Nunc International, Rochester, NY) and stained with Rhodamine phalloidin (Molecular probes) for F-actin or antibodies to Snail (Cell Signaling) after 24 hours. Briefly, for F-actin staining, cells were fixed in 4% paraformaldehyde, permeabilized in 0.1% Triton-X100 and incubated with Rhodamine phalloidine (1∶100 dilution) for 30 min. For the staining with Snail antibody, cells were fixed in 4% paraformaldehyde for 15 min, incubated with 5% normal goat serum in PBS/Triton-X100 for 60 min, with primary antibody at 4°C overnight, and in Alexafluor 568 fluochrome-conjugated secondary antibody for 1 hr at room temperature. The slides were mounted with vectashield mounting media (Vector) and viewed under an Olympus Fluoview FV1000 confocal fluorescence microscope.

### Animal Studies

Four-week-old female BALB/c and athymic nude mice (obtained from Harlan Sprague Dawley, Inc. Indianapolis, IN) were used for *in vivo* animal experiments. The animals were housed under specific pathogen free conditions. We investigated the effects of doxorubicin and/or TβRI-KI treatment on orthotopic xenograft tumors formed by murine 4T1 or human MDA-MB-231 breast cancer cell lines, and also on their subsequent spontaneous lung and/or bone metastasis. Murine breast cancer 4T1 cells (0.1×10^6^) and human breast cancer EGFP expressing MDA-MB-231 cells (1.0×10^6^) were inoculated at the both inguinal mammary fat pad of syngeneic BALB/c mice and athymic nude mice respectively. In the study with 4T1 xenograft model, animals were treated with Doxorubicin (once a week via intraperitoneal (i.p.) or intravenous (i.v.) injection in two doses of 4 mg/kg and 8 mg/kg animal body weight respectively) and TβRI-KI (every alternative day via i.p. injection of 1 mg/kg body wt) either alone or in combination in two sets of experiment. In the first experiment, treatment started immediately after tumor cell inoculation and in the second experiment, treatment started when the average tumor volume per mouse was between 60–70 mm^3^. In the study with MDA-MB-231 cells, treatment of doxorubicin and TβR1-KI with similar doses as described above was started when the average tumor volume was between 80–100 mm^3^/mouse. Placebo control group received PBS every alternate day. In both studies, animals were ranked in closely matched groups according to their tumor volumes. Tumor sizes were measured twice weekly with a caliper, and the tumor volumes were calculated with the equation V = (L×W^2^)×0.5, where L is length and W is width of a tumor. In all studies, animals were sacrificed after 21 days of treatment.

### In vivo spontaneous lung and bone metastasis assay

After the termination of the all in vivo experiments, lungs were excised and metastatic cancer cell colonies were visually observed. In the 4T1 study, lungs were fixed in Bouin's solution (Sigma) and surface lung nodules were counted. In the MDA-MB-231 study, EGFP expressing green lung metastatic colonies were observed and counted under a fluorescence microscope followed by 10% buffered formalin fixation. All lung specimens were embedded in paraffin and stained with hematoxylene and eosin. To analyze spontaneous bone metastasis in the 4T1 model, bone tissues (tibia and femora) were fixed in 10% neutral buffered formalin (Fisher Scientific) for 48 hours at room temperature, decalcified in 10% EDTA and embedded in paraffin. Sections were stained with hematoxylin, eosin, orange G and phloxine. The tumor burden in femora and tibia was examined microscopically.

### Statistical analysis

Student t tests were used for the comparison between two mean values. For the comparison of more than two mean values we used one-way analysis of variance (ANOVA) followed with Newman-Keuls Multiple Comparison Test. All statistical analysis was performed using Prism version 3.03 software (GraphPad Software Inc.).

## Supporting Information

Figure S1 TGFβ and doxorubicin stimulated invasion of murine and human breast cancer cells. In a Boyden chamber invasion assay, 50,000 murine breast cancer 4T1 cells (A) and human breast cancer MDA-MB-231 cells (B) in serum free medium were placed inside the insert of an invasion chamber coated with matrigel and treated with doxorubicin (100 nM) or TGFβ1 (5 ng/ml in A and 1 ng/ml in B) for 18 hours and 24 hours respectively. Lower chamber contained the complete medium with 10% serum as chemoattractant. Stained membranes were counted under a microscope. Results are the mean+SEM of invasive cell numbers in three fields of observation at 100× magnification in A and total number of invasive cells in B.
(0.09 MB TIF)

Figure S2 Doxorubicin treatment enhanced TGFβ signaling in murine and human breast cancer cells. A. The murine 4T1 and human MDA-MB-231 breast cancer cells were treated with doxorubicin (25 nM) or TGFβ1 (2 ng/ml) in the presence or absence of TGFβ antagonist TbRI-KI (500 nM) for 24 hours. The cell extracts were used for Western blotting analysis to measure the levels of phosphorylated Smad3 (P-Smad3). B. Mink lung epithelial cells stably transfected with TGFβ-responsive PAI-1 promoter-luciferase reporter construct were plated in a 96-well plate and treated with doxorubicin or TGFβ1 (0.5 ng/ml) for 24 hr. Luciferase activity was measured in cell lysate. Data are mean±SEM of 3 wells.
(0.09 MB TIF)

Figure S3 Doxorubicin induced EMT in human breast cancer MDA-MB-231 cells. MDA-MB-231 cells were treated with TGFβ1 (2 ng/ml) or doxorubicin (25 nM) in the presence or absence of TbRI-KI (500 nM) for 7 days in an 8-well Lab-Tek chambered coverglass (Nunc International, Rochester, NY). Cells were stained with Rhodamine phalloidin (Molecular probes) and F-actin cytoskeleton was viewed under an Olympus Fluoview FV1000 confocal fluorescence microscope.
(0.28 MB TIF)

Figure S4 Prolonged treatment of 4T1 cells with TGFβ1 enhanced formation of mammospheres. 4T1 cells were treated with 0.05 or 0.1 ng/ml of TGFβ1 for 12 days with medium change every alternate day. Cells were harvested and passed through a 40 µm nylon membrane to obtain a single cell suspension. Cells were plated (2,000 cells per well) in a 24-well ultra low attachment plate in DMEM-F12 mammosphere culture medium. The number of large mammospheres (>100 µm in diameter) obtained from 2,000 cells with or without TGFβ1 treatment were counted and presented in Panel A. The representative photos of mammosphere are shown in Panel B.
(0.10 MB TIF)
