# Supplementary figures and images for: Doxorubicin in Combination with a Small TGFβ Inhibitor: A Potential Novel Therapy for Metastatic Breast Cancer in Mouse Models
Source: PLoS One. 2010 Apr 28;5(4):e10365. doi: 10.1371/journal.pone.0010365 (PMC2860989; doi:10.1371/journal.pone.0010365)

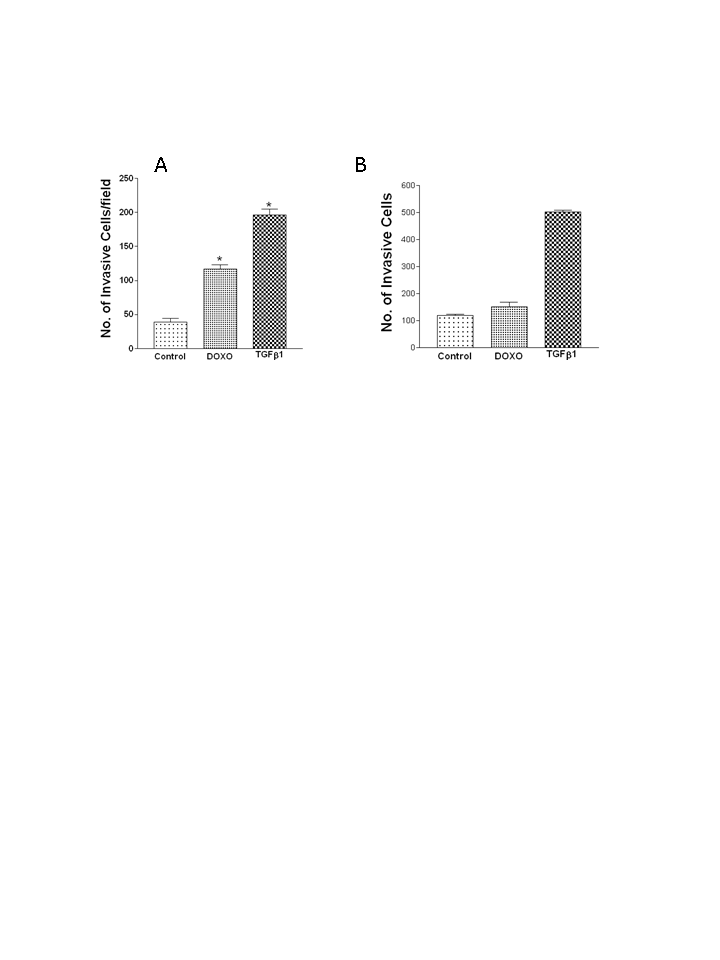

Supplement: Figure S1 — TGFβ and doxorubicin stimulated invasion of murine and human breast cancer cells. In a Boyden chamber invasion assay, 50,000 murine breast cancer 4T1 cells (A) and human breast cancer MDA-MB-231 cells (B) in serum free medium were placed inside the insert of an invasion chamber coated with matrigel and treated with doxorubicin (100 nM) or TGFβ1 (5 ng/ml in A and 1 ng/ml in B) for 18 hours and 24 hours respectively. Lower chamber contained the complete medium with 10% serum as chemoattractant. Stained membranes were counted under a microscope. Results are the mean+SEM of invasive cell numbers in three fields of observation at 100× magnification in A and total number of invasive cells in B. (0.09 MB TIF) [file pone.0010365.s001.tif]

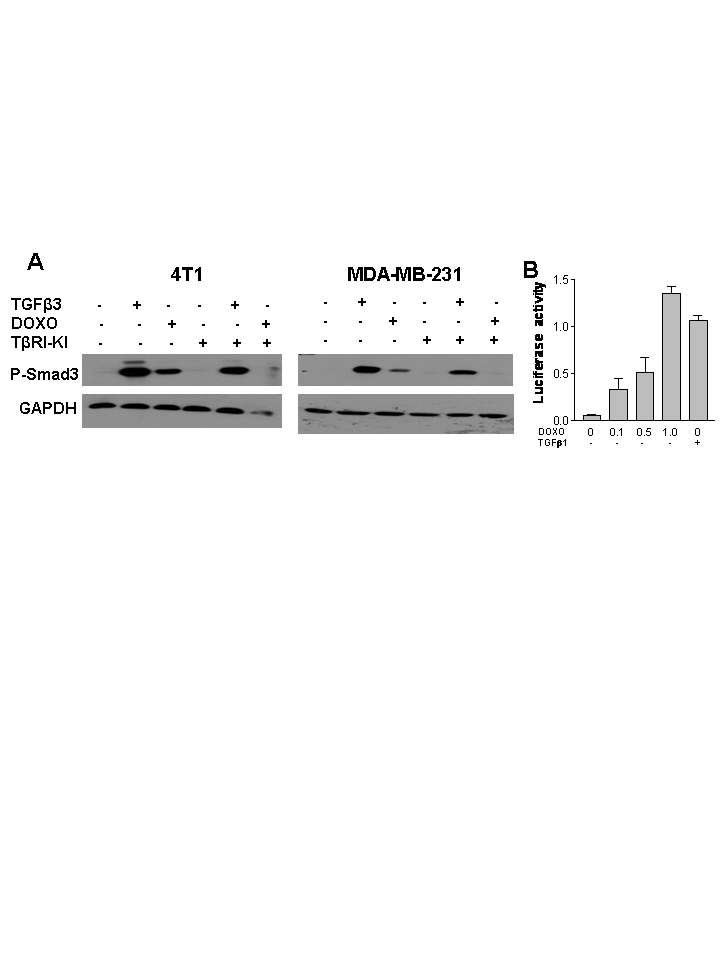

Supplement: Figure S2 — Doxorubicin treatment enhanced TGFβ signaling in murine and human breast cancer cells. A. The murine 4T1 and human MDA-MB-231 breast cancer cells were treated with doxorubicin (25 nM) or TGFβ1 (2 ng/ml) in the presence or absence of TGFβ antagonist TbRI-KI (500 nM) for 24 hours. The cell extracts were used for Western blotting analysis to measure the levels of phosphorylated Smad3 (P-Smad3). B. Mink lung epithelial cells stably transfected with TGFβ-responsive PAI-1 promoter-luciferase reporter construct were plated in a 96-well plate and treated with doxorubicin or TGFβ1 (0.5 ng/ml) for 24 hr. Luciferase activity was measured in cell lysate. Data are mean±SEM of 3 wells. (0.09 MB TIF) [file pone.0010365.s002.tif]

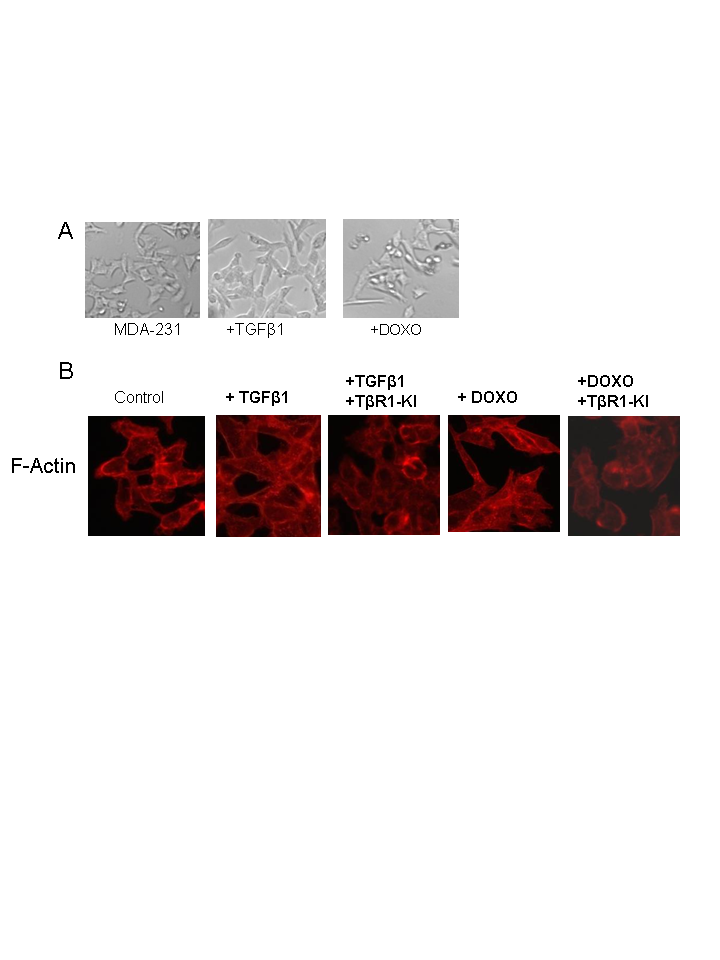

Supplement: Figure S3 — Doxorubicin induced EMT in human breast cancer MDA-MB-231 cells. MDA-MB-231 cells were treated with TGFβ1 (2 ng/ml) or doxorubicin (25 nM) in the presence or absence of TbRI-KI (500 nM) for 7 days in an 8-well Lab-Tek chambered coverglass (Nunc International, Rochester, NY). Cells were stained with Rhodamine phalloidin (Molecular probes) and F-actin cytoskeleton was viewed under an Olympus Fluoview FV1000 confocal fluorescence microscope. (0.28 MB TIF) [file pone.0010365.s003.tif]

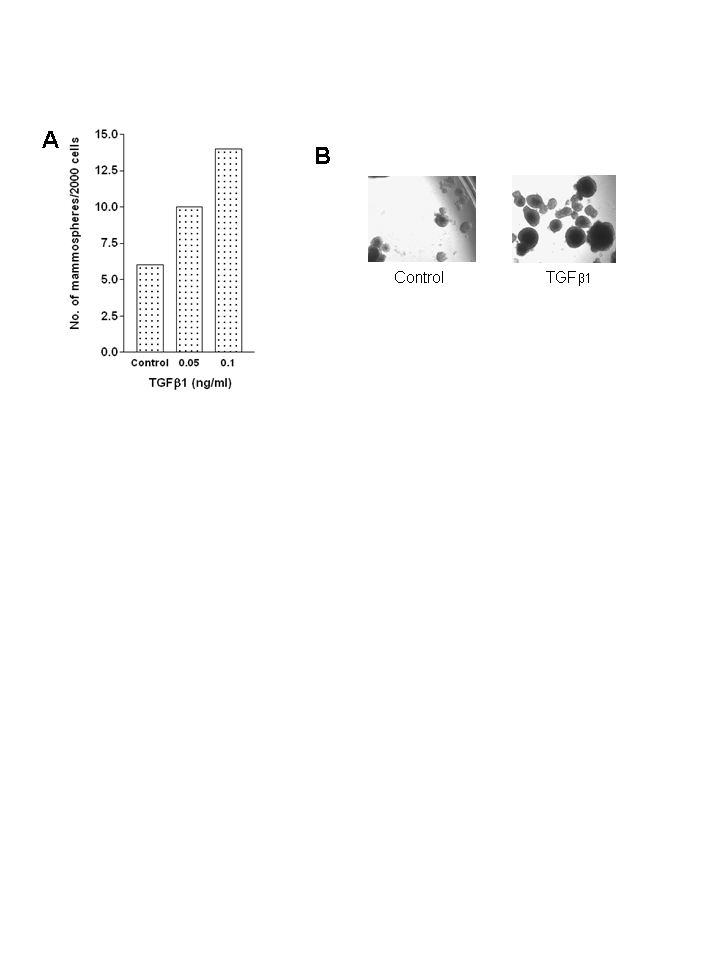

Supplement: Figure S4 — Prolonged treatment of 4T1 cells with TGFβ1 enhanced formation of mammospheres. 4T1 cells were treated with 0.05 or 0.1 ng/ml of TGFβ1 for 12 days with medium change every alternate day. Cells were harvested and passed through a 40 µm nylon membrane to obtain a single cell suspension. Cells were plated (2,000 cells per well) in a 24-well ultra low attachment plate in DMEM-F12 mammosphere culture medium. The number of large mammospheres (>100 µm in diameter) obtained from 2,000 cells with or without TGFβ1 treatment were counted and presented in Panel A. The representative photos of mammosphere are shown in Panel B. (0.10 MB TIF) [file pone.0010365.s004.tif]
